# The HIV matrix protein p17 induces hepatic lipid accumulation via modulation of nuclear receptor transcriptoma

**DOI:** 10.1038/srep15403

**Published:** 2015-10-15

**Authors:** Barbara Renga, Daniela Francisci, Adriana Carino, Silvia Marchianò, Sabrina Cipriani, Maria Chiara Monti, Rachele Del Sordo, Elisabetta Schiaroli, Eleonora Distrutti, Franco Baldelli, Stefano Fiorucci

**Affiliations:** 1 Department of Surgical and Biomedical Sciences, Section of gastroenterology, University of Perugia, Perugia, Italy; 2 Department of Medicine, Section of Infectious diseases, University of Perugia, Perugia, Italy; 3 Department of Biomedical and Pharmaceutical Sciences, University of Salerno, Fisciano, Italy; 4 Department of Experimental Medicine and Biochemical Sciences, Section of Anatomic Pathology and Histology, University of Perugia, Perugia, Italy; 5 Hospital S.M. Misericordia, Perugia, Italy

## Abstract

Liver disease is the second most common cause of mortality in HIV-infected persons. Exactly how HIV infection *per se* affects liver disease progression is unknown. Here we have investigated mRNA expression of 49 nuclear hormone receptors (NRs) and 35 transcriptional coregulators in HepG2 cells upon stimulation with the HIV matrix protein p17. This viral protein regulated mRNA expression of some NRs among which LXRα and its transcriptional co-activator MED1 were highly induced at mRNA level. Dissection of p17 downstream intracellular pathway demonstrated that p17 mediated activation of Jak/STAT signaling is responsible for the promoter dependent activation of LXR. The treatment of both HepG2 as well as primary hepatocytes with HIV p17 results in the transcriptional activation of LXR target genes (SREBP1c and FAS) and lipid accumulation. These effects are lost in HepG2 cells pre-incubated with a serum from HIV positive person who underwent a vaccination with a p17 peptide as well as in HepG2 cells pre-incubated with the natural LXR antagonist gymnestrogenin. These results suggest that HIV p17 affects NRs and their related signal transduction thus contributing to the progression of liver disease in HIV infected patients.

HIV infected patients experience a wide array of adverse metabolic complications and comorbidities^1^,^2^. The most common of these metabolic disfunctions are serum dyslipidemias, changes in body fat distribution, insulin resistance and diabetes mellitus type 2^3^,^4^,^5^,^6^ and therefore the clinical management of HIV infection is focusing on managing these comorbidities and drug toxicities^7^. Although most of the attention in recent years has been on derangements in glucose and lipid metabolism and the associated risk for cardiovascular morbidity, there is also increasing risk to the development of long-term liver dysfunction.

Nonalcoholic fatty liver disease (NAFLD), fat deposition in the liver not caused by chronic alcohol abuse, is an increasingly recognized in Western countries coincident with the obesity pandemic^8^,^9^. Patients with chronic HIV infection are at increased risk of NAFLD evolving into nonalcoholic steatohepatitis (NASH), given the fact that such patients may experience significant metabolic disorders, chronic inflammation, and coinfection with hepatitis viruses. In addition, the use of some nucleoside reverse transcriptase inhibitors (NRTIs) used in the treatment of HIV infection associates with hepatic dysfunction triggered by mitochondrial toxicity^10^,^11^,^12^. HIV infection itself is a potential risk factor for fatty liver disorders^13^.

The HIV matrix protein p17 is a multifunctional protein involved in viral replication including the process of nuclear import of the HIV genome or the targeting of Pr55Gag proteins at plasma membrane^14^. HIV-1-infected cells release significant amounts of virion-free p17. Circulating p17 protein, which is detected in the plasma of HIV-1-infected persons at nanomolar concentrations^15^,^16^, exerts its effects not only in immune cells directly involved in AIDS pathogenesis (i.e. T lymphocytes)^17^, but also in other immune cells (i.e. monocytes)^14^ and in other cell types (i.e. hepatic stellate cells)^18^. The effect exerted by the p17 protein are mediated by activation of a putative p17 receptor, i.e. CXCR2, although p17 binds also to heparan sulphate side chains of syndecan-2 proteoglycan^18^,^19^,^20^. To date, amongst the HIV proteins, none of them has been proved to be able to accelerate the course of liver disease.

Nuclear hormone receptors (NRs) are a family of DNA-binding transcription factors, which regulate a broad spectrum of physiological processes including cell cycle, cellular metabolism, organ homeostasis and embryonic development^21^. NRs and their coregulators play important roles in nonalcoholic fatty liver diseases^22^. Thus, the bile acid sensor, Farnesoid X receptor (FXR) has emerged in recent years as a master regulator of lipid homeostasis in the liver. This conclusion became from the observation that FXR-knockout mice on a high-fat diet exhibit hyperlipidemia and massive hepatic steatosis, as well as necroinflammation and fibrogenesis^23^,^24^. FXR induces expression of genes that promote triglyceride clearance and mitochondrial fatty acid β-oxidation, as well as suppression of lipogenic gene transcription^25^,^26^. In addition to FXR also the liver X receptor (LXR) has emerged as an important regulator of hepatic lipogenesis^27^,^28^,^29^. Contrary to FXR, liver activation of LXR stimulates hepatic lipogenesis through transcriptional regulation of sterol regulatory element-binding protein (SREBP)-1c, acetyl-CoA carboxylase, stearoyl-CoA desaturase-1 and fatty acid synthase leading to increased fatty acid biosynthesis and plasma triglycerides^27^,^28^,^29^.

In the present study we have characterized the expression profile of NRs and their related coregulators in HepG2, a human hepatoma cell line, exposed to the HIV matrix protein p17. We found that p17 increases the expression and transcriptional activity of LXR and its coactivator MED1. Furthermore, p17 increases the hepatic lipid accumulation via activation of LXR/SREBP1c lipogenic pathway. Present findings describe how a viral factor highjacks mammalian regulatory pathways causing liver injury.

## Methods

### Chemicals

Fludarabine (a STAT-1 inhibitor) and 5,15 DPP (a STAT-3 inhibitor) were purchased from Sigma (St. Louis, MO, USA). Recombinant HIV-p17 protein was provided by Medestea (Torino, Italy). Gymnestrogenin was kindly provided by Prof. Angela Zampella (University of Naples, Italy – angela.zampella@unina.it).

### Cell culture

HepG2 cells, an immortalized human hepatocarcinoma cell line, were cultured at 37 °C in an atmosphere of 5% CO_2_ in E-MEM medium containing 10% fetal bovine serum, antibiotics (100 U/ml penicillin, 100 U/ml streptomycin) and 1% glutamine.

Primary human hepatocytes were purchased from Innoprot S.L. and cultured in Hepatocyte medium (cat. P60109) containing 5% fetal bovine serum, antibiotics (100 U/ml penicillin, 100 U/ml streptomycin) and 1% hepatocyte growth supplement and kept at 37 °C in a humidified atmosphere of 5% CO2.

### Isolation, culture and stimulation of mouse primary hepatocytes

Hepatocytes were isolated from livers obtained by C57BL6 mice according to the method of Severgnini *et al.*^30^ with minor modifications. Methods were carried out in accordance with the approved guidelines. Experimental protocol was approved by ethical committee of University of Perugia. In brief, livers were perfused with Hank’s Balanced Salt Solution (HBSS) containing EDTA 0.5 mM pH = 8 for 6 minutes, followed by collagenase type I (Sigma Aldrich) 0.8 mg/ml in DMEM for 6 minutes (flow rate 5 ml/min). At the end of the perfusion livers were collected, the liver sacs were cut to release the hepatocytes, 35 ml of medium were added and then the suspension was passed through a cell strainer. Cells were centrifuged at 50 g for 1 minutes, washed for two times with PBS w/o calcium and magnesium and resuspended in culture media (DMEMF12 with penicillin/streptomycin, 5% FCS, 2 mM glutamine, 100 nM insulin, 100 nM dexamethasone). Hepatocytes were then plated in collagen-coated 6 well plates at a density of 1 × 10^5^ cells/well in a 95% air and 5% CO_2_ and stimulated for 18 hours with 2 and 10 μg/ml of p17 (equivalent to 134 and 670 nM respectively).

### Oil red O staining

After 7 days treatment with p17 (μg/ml) cells were washed three times with iced PBS and fixed with 4% paraform for 30 minutes. After fixation, cells were washed three times and stained with Oil Red O solution (working solution, 0.5 g Oil Red O powder dissolved in 60% ethanol) for 15 min at room temperature. Cells were washed again with phosphate-buffered saline (PBS) to remove unbound staining. To quantify Oil Red O content levels, dimethyl sulfoxide was added to each sample; after shaking at room temperature for 5 min, the density of samples were read at 510 nm on a spectrophotometer.

### RNA extraction and quantitative Real-Time PCR

Total RNA was extracted from HepG2 cells, human or murine primary hepatocytes using 1 ml Trizol Reagent (Life Technologies) and cDNA synthesis was carried out from 1 μg RNA using the reverse transcriptase enzyme SSII (Life Technologies) according to manufacturer’s protocol. Ten nanograms cDNA was used in 25 μl final volume reaction of Real-Time PCR contained the following reagents: 0.2 μmol/L of each primer and 12.5 μl of 2X KAPA SYBR master mix (Kapa Biosystems). All reactions were performed in triplicate and the thermal cycling conditions were: 2 min at 95 °C, followed by 40 cycles of 95 °C for 10 seconds, 60 °C for 45 seconds in iCycler iQ instrument (Biorad, Milan, Italy). The results of Real-Time PCR were normalized with the housekeeping gene GAPDH according to the C_t_ method. In brief, the relative mRNA expression has been first calculated as ΔC_t_ (the difference between the C_t_ of the investigated gene and the C_t_ of GAPDH for each sample (es. control sample and treated sample). Than, ΔΔC_t_ is calculated as the difference between the ΔC_t_ of treated sample and the ΔC_t_ of control sample. For control sample the ΔΔC_t_ is calculated as the difference between the ΔC_t_ of control sample and itself. Finally, the relative mRNA level of the investigated gene is expressed as 2^–(ΔΔCt)^. All PCR primers were designed using software PRIMER3-OUTPUT using published sequence data from the NCBI database. Sequences of forward and reverse primers were: hGAPDH: gaaggtgaaggtcggagt and catgggtggaatcatattggaa; hLXRα: caaccctgggagtgagagtatc and atagcaatgagcaaggcaaact; hPPARγ: gctggcctccttgatgaata and ttgggctccataaagtcacc; hCXCR2: gctctgactaccacccaacc and gctgggcttttcacctgtag; hSyndecan-2: gatgacgatgactacgcttctg and aggtgactttgtctgagcaggt; hSREBP1c: gcaaggccatcgactacatt and ggtcagtgtgtcctccacct; hFAS: cgaagaggcgtgccctgagct and gccgtagttgctctgtcccg; hCH25H: tacaacttcccttggtccactc and tgtcccagtgtgtaaagtacgg; hBRD8: tgatacatggtggggagataca and ctacccctgatcgaaaacactc; hKAT5: gaaccaggacaacgaagatga and ggggaactggatcttctttagg; hMED1: accaagatgaaacctcaaggaa and ataggggacttggctttagagg; hDDX5: gcacaagaggtggaaacataca and actccaaccatatccaatccac; hSCD-1: gcagaatggaggagataagtt and aatcaaagtgatcccatacag; hABCA1: gcttgggaagatttatgacagg and aggggatgattgaaagcagtaa; mGAPDH: ctgagtatgtcgtggagtctac and gttggtggtgcaggatgcattg; mLXR: gcaggaccagctccaagtag and ggctcaccagcttcattagc; mSREBP1c: gatcaaagaggagccagtgc and tagatggtggctgctgagtg; mFAS: tgggttctagccagcagagt and accaccagagaccgttatgc; hPEPCK: aggcggctgaagaagtatga and ggatgggcactgtgtctctt; hPTGDS: acctactccgtgtcagtggtg and gacaatggtatcctctgtgaagc; hPDK4: atatcctcccgacccaattagt and tctatgattccttgtgccattg; hFABP1: aggaatgtgagctggagacaat and aatgtcacccaatgtcatggta; hESR1: accaatgacaagggaagtatgg and tggctggacacatatagtcgtt.

### Microarray analysis

Total RNA from HepG2 cells left untreated or stimulated 18 hours with p17 (2 μg/ml) was extracted with Trizol reagent (Invitrogen) and reverse transcribed with Superscript-II reverse transcriptase (Invitrogen) following the manual instructions. 10 ng cDNA was pipetted in each well of a 96 well PCR array plate (Human Nuclear Receptors and Coregulators RT^2^ *Profiler*™ PCR Array - http://www.sabiosciences.com/rt_pcr_product/HTML/PAHS-056 A.html - Superarray Bioscience, Frederick, MD, USA) and amplified following the manual instructions. Genes selected for PCR analysis encode several classes of nuclear receptors and co-regulators of transcription, including co-activators and co-repressors. PCR analysis was carried out with the on-line software *RT*^2^ *Profiler PCR Array Data Analysis*(http://pcrdataanalysis.sabiosciences.com/pcr/arrayanalysis.php)

### Western blotting

To investigate the effect of p17 on phosphorylation status of STAT1 and STAT3 total lysates were prepared by solubilization of cells in E1A lysis buffer (250 mM NaCl, 50 mM Hepes pH 7.0, 0.1% NP40, 50 mM EDTA) containing phosphatase and protease inhibitors. To investigate the effect of p17 on total protein expression CXCR2, syndecan-2 and tubulin, proteins were prepared for electro phoresis by boiling in 1X SDS Sample buffer (50 mM Tris HCl pH 6.8, 2.5% beta mercaptoethanol, 2% SDS, 10% glycerol). Following transfer to nitrocellulose membranes (Bio-Rad) proteins were detected with the following primary antibodies: CXCR2 (Santa Cruz – sc-7304), syndecan-2 (Santa Cruz – sc-365624), a-tubulin (Sigma – T 6074), phospho STAT1(tyr701) (Cell Signaling - #9171), total STAT1 (Cell Signaling - #9172), phospho STAT3(tyr705) (Santa Cruz – sc-7993) and total STAT3 (Santa Cruz – sc-8019). Primary antibodies were detected with the horseradish peroxidase (HRP)-labeled secondary antibodies. Proteins were visualized by SuperSignal West Dura Extended Duration Substrate (Thermo Fisher Scientific Inc.) according to the manufacturer’s instructions.

### Vaccination protocol

The HIV matrix protein p17 was immune-neutralized by incubation with sera from an HIV infected patient enrolled in a Phase 1 study designed to investigate the safety and immunogenicity of recombinant p17 peptide in HIV. The therapeutic vaccination was performed using a 20 amino acids peptide, named AT20-KLH (SGGELDRWEKIRLRPGGKKK). Methods were carried out in accordance with the approved guidelines. The vaccination protocol n. MED-AT20-001 Eudract Number 2008-001465-29 had been approved by the Ethical committee of Regione Umbria (Italy) on June 25, 2010 authorization n. 1558/10. Authorization for collecting and using blood samples from HIV infected persons for *ex vivo* testing was also granted by the ethical committee of Regione Umbria (Italy) on July 22, 2010 (authorization number CEAS 1654/20). An informed written consent was obtained from each participant to the study.

### Cholesterol and 25-OH-cholesterol determination

Sample preparation. Stock solutions of Cholesterol and 25-hydroxy Cholesterol were separately prepared at 5 mg/mL using MeOH as solvent. Five dilutions were obtained mixing 1 ng, 10 ng, 100 ng, 1 μg and 10 μg of Cholesterol and 25-hydroxy Cholesterol in 10 μL of MeOH. 100 μL of PBS at pH of 7.5 and 5 μL of the enzyme Cholesterol Oxidase from Streptomyces *sp.* at 0.2 mg/mL in water were added to each sample for 1 h at 37 °C. Later on, 200 μL of MeOH, 15 μL of glacial acetic acid and 15 mg of Girard T reagent (diluted in 20 μL of water) were added and kept in the dark at room temperature overnight (final volume of 350 μL) as reported^31^. HepG2 cells pellets were treated with 500 μL of pure EtOH, alternatively vortexed and sonicated for 30 min. All samples were then centrifuged for 1 h at 13300 g and clear surnatants were separated from insoluble materials and dried under N_2_. Each sample was diluted in 10 μL of MeOH and treated as reported above. Liquid chromatography-mass spectrometry analysis. For LC–MS/MS analysis, chromatographic separation was carried out on the HPLC–MS system LTQ XL Thermo Scientific equipped with Accelera 600 Pump and Accelera AutoSampler system. The mixture was separated on a JUPITER C18 column from Phenomenex (150 × 2.00 mm) and the column flow rate was set at 150 μl/min. Samples were separated using a acetonitrile-metanol-aqueous gradient. Mobile phase A was water/MeOH/ACN at 50/33.3/16.7% in 0.1% FA, mobile phase B was MeOH/ACN at 66.6/33.4% in 0.1% TFA. The gradient started at 35% B and increased to 95% B in 15 min, kept at 95% B for 10 min then decreased to 35% B in 1 min and kept at 35% B for 10 min. ESI was performed in positive ion mode, the ion source temperature was set at 280 °C. The MS/MS detection was operated using a collision energy of 40 (arbitrary units). 25-Hydroxy-Colestherol modified by GT reagent gave a positive ion at m/z of 514.5 at 17.0 min and MS/MS analysis gave fragments at m/z of 455.4, 437.4, 427.4, 163.1, 151.1, 135.1, 123.1. Colestherol modified by GT reagent gave a positive ion at m/z of 498.5 at 22.7 min and MS/MS analysis gave fragments at m/z of 439.4, 411.4, 163.1, 151.1, 135.1, 123.1.

### Chromatin Immunoprecipitation

Serum starved HepG2 cells (10 × 10^6^) were left untreated or stimulated with p17 (2 μg/ml) 18 h. After stimulation cells were cross-linked with 1% formaldehyde 10′ at room temperature and then the reaction terminated by the addition of glycine to a final concentration of 125 mM. Cells were washed in ice-cold PBS and lysed with 500 μl ChIP lysis buffer (1% SDS, 10 mM EDTA, and 50 mM Tris–HCl, pH 8) containing 10 μM PMSF and protease inhibitor cocktail (Sigma), sonicated and centrifuged at 13000 rpm 10′ at 4 °C. Fifty microliters of each supernatant (Input DNA) were reverse-cross-linked by the addition of 150 μl Elution buffer (1% SDS and 0.1 M NaHCO3) and 8 μl NaCl 5 M and by heating the mixture to 65 °C for 4 h. DNA was recovered from input by proteinase K treatment at 65 °C for 1 h followed by phenol/chloroform (1:1) extraction, ethanol precipitation and dissolved into 50 μl of molecular biology grade water. Thus, input DNA was spectrophotometrically quantified and 40 μg chromatin was diluted with ChIP dilution buffer (0.01% SDS, 1% Triton-X-100,1.2 mM EDTA pH 8.0, 16.7 mMTris–HCl pH 8.0, 167 mM NaCl) containing protease inhibitors and 20 μl of ChIP lysis buffer equilibrated Protein A Sepharose (Amersham Bioscience)/Salmon Sperm DNA/1% BSA. After mixing at 4 °C for 1 h, the mixtures were centrifuged at 1000 rpm for 1 min to obtain pre-cleared supernatants. Pre-cleared supernatants were immunoprecipitated overnight at 4 °C with 4 μg specific antibodies: anti-STAT1 (Cell Signaling) anti-STAT3 (Santa Cruz) or anti IgG (SA1-36098-Pierce). Immunoprecipitates were washed sequentially with low-salt wash buffer (0.1% SDS, 1% Triton-X-100, 2 mM EDTA pH 8.0, 20 mM Tris–HCl pH 8.0, 150 mM NaCl) and then with high-salt wash buffer (0.1% SDS, 1% Triton-X-100, 2 mM EDTA pH 8.0, 20 mM Tris–HCl pH 8.0, 500 mM NaCl). DNA was eluted by addition of 250 μl Elution buffer and the cross-linking reactions were reversed by heating the mixture to 65 °C overnight. The DNA was recovered from immunoprecipitated material by proteinase K treatment at 65 °C for 1 h followed by phenol/chloroform (1:1) extraction, ethanol precipitation and dissolved into 50 μl of molecular biology grade water. Five microliters of chromatin was used for quantitative real-time PCR. Raw data analysis was performed as follows: ΔCt was calculated versus the input DNA concentration; ΔΔCt was versus unstimulated cells immunoprecipitated with the anti-IgG antibody (experimental condition set as 1.0); the relative expression was calculated as 2−(ΔΔCt). The sequences of primers used for the amplification of the LXR promoter (–276/–227 bp from ATG) were as follows: AGGTCAATCCTGAATCCA and AGTCTGCGCTTCGTTACCTT.

### Immunohistochemistry analysis

Immunohistochemical analysis of HIV p17 protein was performed in primary human hepatocytes left not treated or stimulated with 2 μg/ml p17 for 18 hours. Cells were fixed in PFA 4% in PBS for 5 min and permeabilized with PBS 0.5% Triton-X100 for 15 min. Endogenous peroxidase was blocked using H_2_O_2_ for 10 min. An anti-HIV p17 mouse monoclonal antibody (ab20851 – Abcam Inc.) was used at a dilution of 1:100 in PBS 6% BSA with an overnight incubation at 4 °C. Cells were then treated for 30 min with 10 μg/ml Biotinylated secondary antibody (Vector Laboratories) and incubated with the ABC reagent (Vector Laboratories) for a further 30 min at room temperature. A DAB substrate (Vector Laboratories) was used as chromogen and Haematoxylin counter-staining was performed.

Immunohistochemistry of human liver biopsies. Human liver tissues used in the present study were obtained from needle biopsy previously performed in HIV patients with NASH and healthy patients without NASH. Methods were carried out in accordance with the approved guidelines. Experimental protocol was approved by ethical committee of University of Perugia. Informed written consent was obtained from all patients. HIV positive patients were in therapy with second generation NRTIs (i.e Kivexa or Truvada) and were HCV and HBV negative. Sections of 4 μm thick were performed by original formalin-fixed, paraffin embedded tissue blocks. Sections were immunostained for the following antibodies: LXRα (Abcam), SREBP1c (Abcam). The primary antibody was detected using a biotin-free polymeric-horseradish peroxidase (HRP)-linker antibody conjugate system (Bond Polymer Refine Detection, Leica) conducted with the Bond III automated immunostainer (Leica).

### Statistical Analysis

All results are expressed as mean 6 standard error (SE). Comparisons of more than two groups were made with a one-way ANOVA with post-hoc Tukey’s test. Differences were considered statistically significant when P was < 0.05.

## Results

### HepG2 cells express CXCR2 and syndecan-2 and their stimulation with p17 modulates nuclear receptors and coregulators gene expression

**Figure 1:**
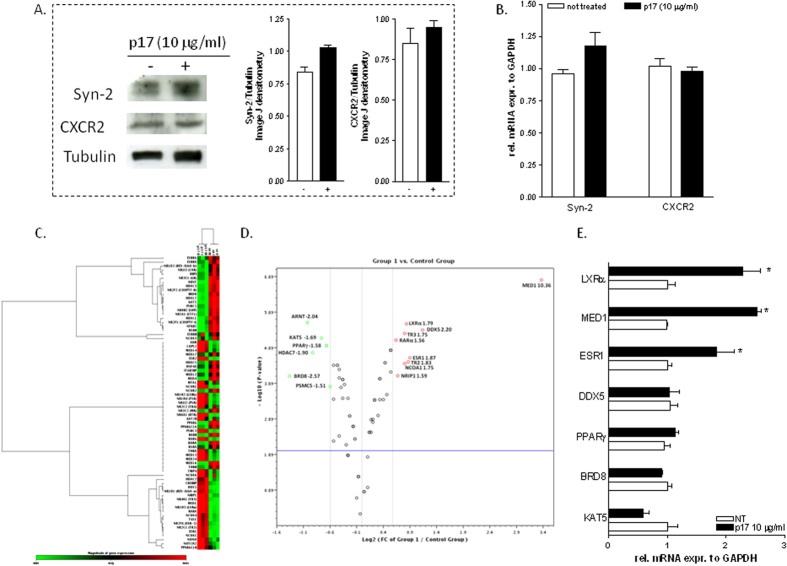
HIV p17 regulates NRs and transcriptional coregulators in HepG2 cells. (**A**) Immunoblot of syndecan-2 (Syn-2), CXCR2, and Tubulin. (**B**) qRT-PCR of syn-2 and CXCR2. Data represent the mean of 4 experiments. Values are normalized relative to GAPDH mRNA and are expressed relative to those of untreated cells, which were arbitrarily set to 1. *P,0.05 versus not treated cells. (**C,D**) Microarray analysis of nuclear receptors and transcriptional coregulators in HepG2 cells stimulated with p17 (10 μg/ml). Out of 84 genes examined, nine genes were upregulated 1.5-fold or greater (LXRα, MED1, DDX5, TR2, TR3, RARα, ESR1, NCOA1, NRIP1) while six genes were down-regulated 1.5 -fold or greater (PPARγ, KAT5, BRD8, PSMC5, HDAC7, ARNT). (**C**) Clustergram and (**D**) Scatter plot. (**E**) Validation of microarray results by RT-PCR showing the relative mRNA expression of LXRα, MED1, ESR1, DDX5, PPARγ, BRD8 and KAT5. Values are normalized relative to GAPDH mRNA and are expressed relative to those of untreated cells, which were arbitrarily set to 1. *P,0.05 versus not treated cells.

Since the HIV matrix protein p17 exerts its pro-inflammatory and pro-fibrogenic effects through the binding with CXCR2 and syndecan-2^17^,^18^, we have first examined whether HepG2 cells, a human immortalized hepatocarcinoma cell line, express these two proteins. As shown in Fig. 1A,B, HepG2 cells express CXCR2 and syndecan-2 as demonstrated by Western blotting and RT-PCR analysis, and the treatment with p17 (10 μg/ml) did not significantly alter the gene or protein expression of these two membrane proteins.

We have next explored the effect of viral protein p17 on mRNA expression of NRs and transcriptional coregulators in HepG2 cells using a real-time PCR-based focused microarrays. A comparison of 84 genes between HepG2 and HepG2 cells primed with p17 is shown in Fig. 1C,D. Out of 84 genes examined, nine genes were upregulated 1.5-fold or greater (LXRα, MED1, DDX5, TR2, TR3, RARα, ESR1, NCOA1, NRIP1) while six genes were down-regulated 1.5 -fold or greater (PPARγ, KAT5, BRD8, PSMC5, HDAC7, ARNT) in HepG2 cells stimulated with p17 (Fig. 1D). Since these results demonstrated that p17 modulates the expression of several NRs (i.e. LXRα, PPARγ and ESR1) and related coregulators (i.e. MED1, BRD8, KAT5, DDX5) which are known to be involved in the regulation of lipid homeostasis we have validated the results obtained with the PCR based focused microarray by a classical RT-PCR designed to detect LXRα and its coactivator MED1^32^, PPARγ and its coactivators BRD8 and KAT5^33^,^34^ and ESR1 and its coactivator DDX5^35^. The results confirmed that the stimulation of HepG2 cells with p17 caused a significant up-regulation of the expression of LXRα and its coactivator MED1 (Fig. 1E, p < 0.05) as well as ESR1 but not that of its coactivator DDX5 (Fig. 1E, p < 0.05). Further on, exposure to p17 failed to down-regulate the mRNA expression of PPARγ and its transcriptional coregulators BRD8 and KAT5 (Fig. 1E).

### Expression of nuclear receptors target genes is increased by p17 treatment

**Figure 2:**
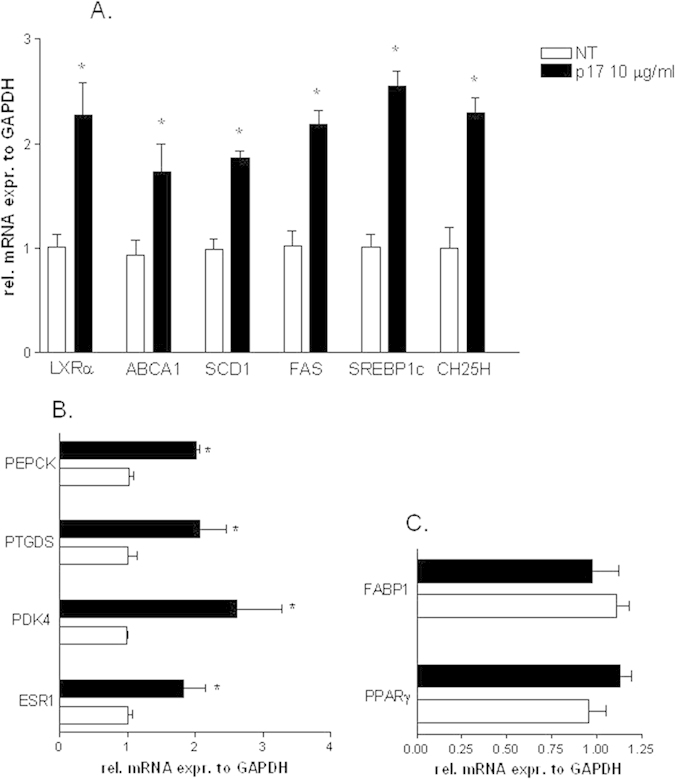
*In vitro* pharmacological evaluation of NRs target genes in HepG2 cells treated with p17. (**A**) Real-time PCR analysis of mRNA expression of LXR and LXR target genes ABCA1, SCD-1, FAS, SREBP1c and CH25H. (**B**) Real-time PCR analysis of mRNA expression of ESR1 and ESR1 target genes PDK4, PTGDS and PEPCK. (**C**) Real-time PCR analysis of mRNA expression of PPARγ and PPARγ target gene FABP1. Values are normalized relative to GAPDH mRNA and are expressed relative to those of untreated cells, which were arbitrarily set to 1. *P,0.05 versus not treated cells.

We have then examined whether the HIV matrix protein p17 regulates canonical functions exerted by LXRα, ESR1 and PPARγ in HepG2. As shown in Fig. 2A, p17 (10 μg/ml) effectively increased the expression of LXRα and its canonical target genes ABCA1, SCD-1, SREBP1c, FAS and CH25OH (Fig. 2A *p < 0.05 vs NT cells). As illustrated in Fig. 2B, p17 induced the expression of both ESR1 and its target genes PDK4, PTGDS and PEPCK (Fig. 2B, *p < 0.05 vs NT cells). In contrast, results shown in Fig. 2C confirmed that p17 has no direct effects on PPARγ and its target gene FABP1 (Fig. 2C).

### Effect of p17 on cholesterol conversion and lipid accumulation in HepG2 cells

**Figure 3:**
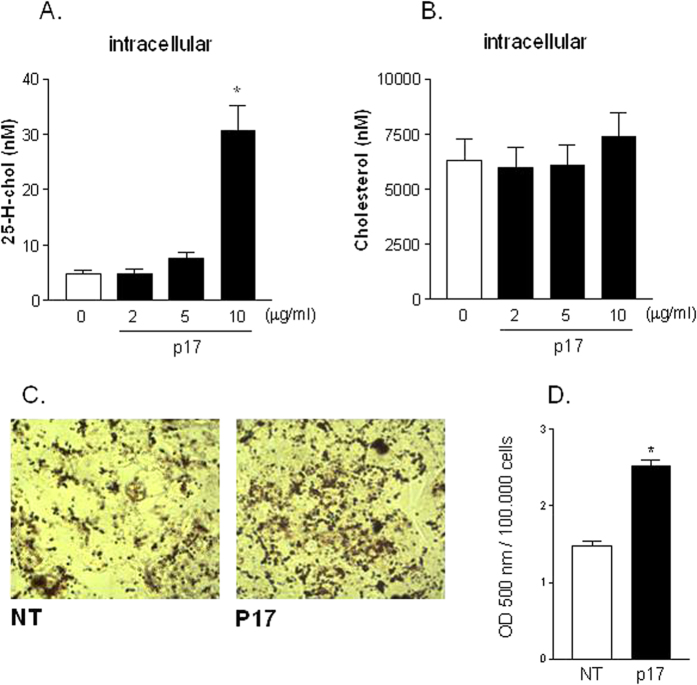
Seven days treatment of HepG2 with p17 increases intracellular content of 25-hidroxicholesterol and induces lipid accumulation. Intracellular content of 25-H-cholesterol (**A**) and total cholesterol (**B**) was measured in HepG2 cells stimulated with escalating doses of p17 (from 2 to 10 μg/ml) for 7 days. (**C**) Observation of lipid accumulation in HepG2 cells treated with 10 μg/ml p17 for 7 days. Representative photomicrographs (40X) of normal or p17 treated cells. (**D**) Oil Red O quantification at 500 nm.

Having shown that p17 induces the relative mRNA expression of cholesterol 25-hydroxylase we have next investigated the intracellular content of both cholesterol and 25-hydroxycholesterol in HepG2 cells exposed to p17. As shown in Fig. 3A,B, incubation of HepG2 cells with escalating doses of p17 (from 2 to 10 μg/ml) caused a significant increase in intracellular content of 25-hydroxycholesterol without altering the intracellular content of total cholesterol (Fig. 3A,B, p < 0.05). In addition, exposure of HepG2 cells to p17 (10 μg/ml) for 7 days increased intracellular lipid content as detected by Oil Red O staining (Fig. 3C) and direct measurement of lipid content (Fig. 3D).

### P17 mediated induction of LXRα occurs via promoter dependent recruitment of STAT transcription factors

**Figure 4:**
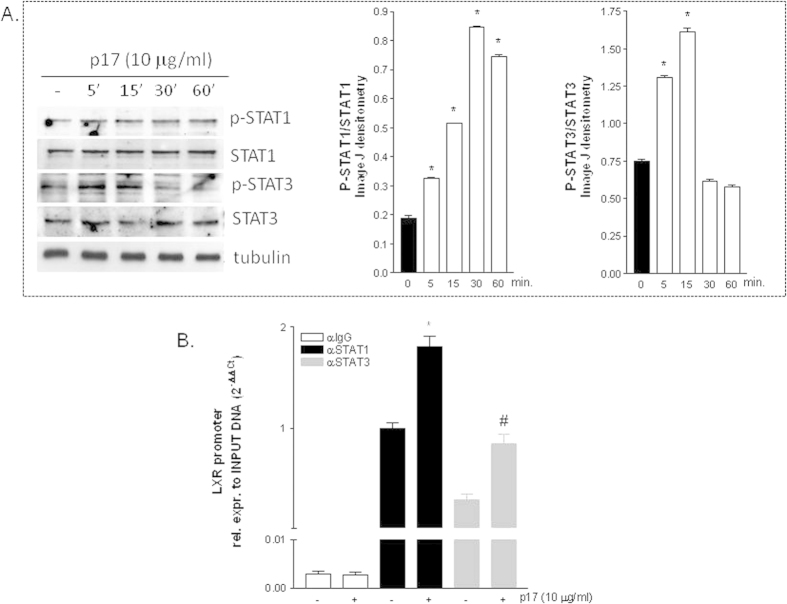
HIV matrix protein p17 activates and recruits STAT proteins to LXR promoter. (**A**) Time course of STAT1 and STAT3 phosphorylation status in HepG2 cells treated with 10 μg/ml of p17 for 5, 15, 30 and 60 minutes. Band densitometry was carried out using the Image J software. (**B**) Chromatin Immunoprecipitation assay was carried out in HepG2 cells left untreated or primed with 10 μg/ml p17 for 18 hours. Real-Time PCR was performed on LXRα promoter as described in materials and methods.

**Figure 5:**
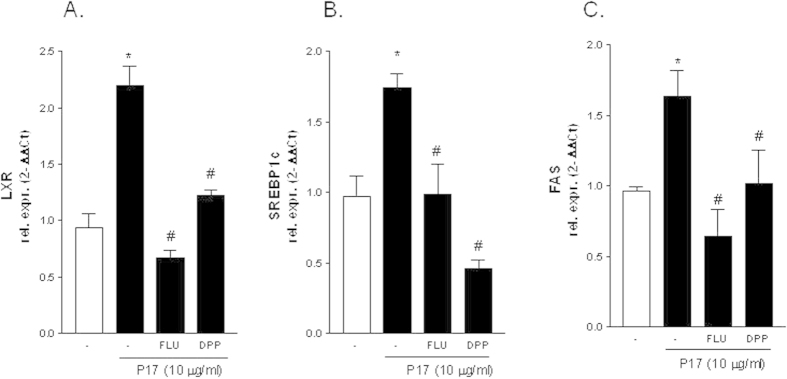
Inhibition of STAT signaling blocks lipogenic effect of p17. HepG2 cells were pre-incubated for 2 h with the STAT1 inhibitor Fludarabine (5 μM) or with the STAT3 inhibitor 5,15 DPP (5 μM) prior to stimulation with 10 μg/ml p17 for 18 hour. qRT-PCR of LXRα (**A**), SREBP1c (**B**) and FAS (**C**). Values are normalized relative to GAPDH mRNA and are expressed relative to those of untreated cells, which were arbitrarily set to 1. *P < 0.05 versus not treated cells. ^#^P < 0.05 versus p17 stimulated cells.

Since in other tissues p17 signals by activating the JaK/STAT pathway^14^,^18^ we have next investigated whether p17 induces the phosphorylation of STAT proteins (i.e. STAT1 and STAT3) in HepG2 cells. Indeed, as illustrated in Fig. 4A, exposure of HepG2 to p17 (10 μg/ml) increased the phosphorylation status of both STAT1 and STAT3 reaching a peak after 5 minutes. In addition, exposure to p17 recruits STAT1 and STAT3 to the LXR promoter, as show in ChIP experiments using quantitative Real-Time PCR performed with primers flanking the LXR promoter region containing the STAT responsive sequence **TTC**AGG**GAA (**Fig. 4B). The relevance of STAT1 and STAT3 in mediating the effect exerted by p17 on LXR was further examined by co-administering HepG2 cells with the STAT1 inhibitor fludarabine or the STAT3 inhibitor 5,15 DPP. Results from these experiments demonstrated that both fludarabine and 5,15 DPP caused a robust reduction of p17 mediated stimulation of LXRα and its target genes SREBP1c and FAS (Fig. 5A–C, p < 0.05).

### The HIV matrix protein p17 activates LXR signaling in primary hepatocytes

**Figure 6:**
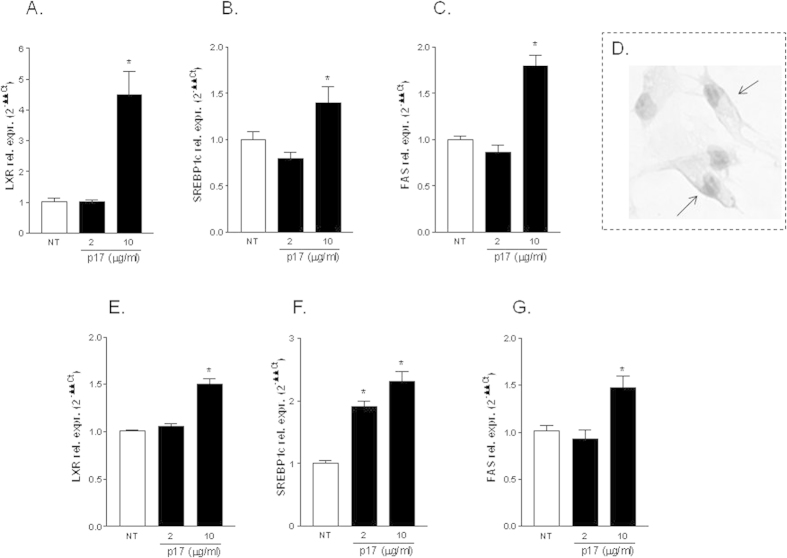
Stimulation of primary hepatocytes with p17 activates LXR signaling pathway. (**A–C**) Human primary hepatocytes were stimulated with two doses of p17 (2 and 10 μg/ml). After stimulation the relative mRNA expression of LXRα (**A**), SREBP1c (**B**) and FAS (**C**) was evaluated by RT-PCR. (**D**) Immunohistochemical staining of p17 on human hepatocytes showing plasma membrane localization of this viral protein. Magnification 40X. (**E–G**) Mouse primary hepatocytes were stimulated with two doses of p17 (2 and 10 μg/ml). After stimulation the relative mRNA expression of LXRα (**A**), SREBP1c (**B**) and FAS (**C**) was evaluated by RT-PCR. Values are normalized relative to GAPDH mRNA and are expressed relative to those of untreated cells, which were arbitrarily set to 1. *P < 0.05 versus not treated cells.

To investigate whether these observation extend to non-transformed hepatocytes, human primary hepatocytes were exposed to escalating doses of p17 (2 and 10 μg/ml) and the relative mRNA expression of LXRα and its target genes (SREBP1c and FAS) assessed by RT-PCR. Results from these experiments demonstrated that the stimulation of primary human hepatocytes with 2 μg/ml p17 failed to regulate LXRα expression/activity while administering these cells with 10 μg/ml significantly increased the expression of LXRα and its target genes (i.e. SREBP1c and FAS) (Fig. 6A–C, *p < 0.05 vs NT cells). Importantly, p17 interacts with CXCR2 and syndecan-2, has demonstrated by immunohistochemical analysis shown in Fig. 6D demonstrating that the HIV p17 localize to the plasma membrane of hepatocytes (Fig. 6D). Similarly to human primary hepatocytes, exposure of mouse primary hepatocyes to 10 μg/ml p17 resulted in a significant upregulation of LXR expression/activity (Fig. 6E,F, *p < 0.05 vs NT cells).

### P17 vaccination as well as LXR antagonism neutralize lipogenic effect of p17

**Figure 7:**
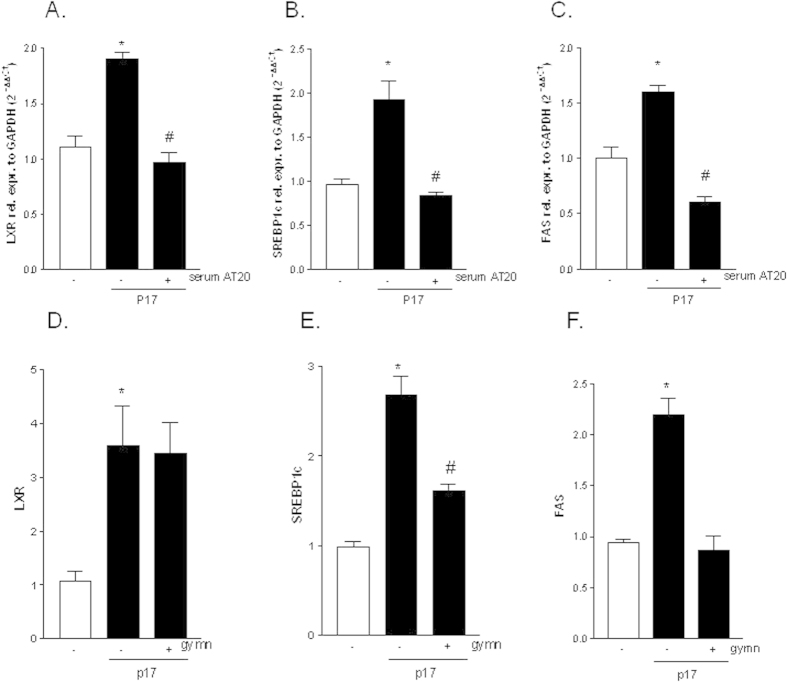
Lipogenic effect of p17 is neutralized with AT20 peptide or LXR antagonism. (**A–C**) Serum starved HepG2 cells were pre-incubated 2 h with the serum taken from an HIV patient vaccinated with a recombinant p17 peptide, diluted 1:100 in culture medium, followed by additional treatment with 10 μg/ml p17 for 18 hours. qRT-PCR of LXRα (**A**), SREBP1c (**B**) and FAS (**C**). (**D–F**) Serum starved HepG2 cells were pre-incubated 2 h with 10 μM LXR antagonist gymnestrogenin, followed by additional treatment with 10 μg/ml p17 for 18 hours. qRT-PCR of LXRα (**D**), SREBP1c (**E**) and FAS (**F**). Values are normalized relative to GAPDH mRNA and are expressed relative to those of untreated cells, which were arbitrarily set to 1. *P < 0.05 versus not treated cells. ^#^P < 0.05 versus p17 stimulated cells. Gymn: gymnestrogenin.

Having shown that the HIV matrix protein p17 regulates the expression of genes involved in hepatic lipid accumulation we next investigated whether these effects were p17 specific leveraging the availability of a serum obtained from an HIV-infected patient vaccinated with p17 peptide AT20. We previously demonstrated that this serum contains neutralizing p17 antibodies^14^,^36^. As illustrated in Fig. 7A–C, exposure of HepG2 cells to p17 neutralizing antibodies retained in the sera of a vaccinated patient completely abrogates the effects of p17 (10 μg/ml) in terms of induction of LXRα and its target genes SREBP1c and FAS (Fig. 7A–C, p < 0.05).

To further confirm the pivotal role of LXR in mediating lipogenic effect exerted by p17 in HepG2 cells we performed an experiment using the recently discovered LXR antagonist gymnestrogenin^37^. Results from this experiment demonstrated that in presence of gymnestrogenin p17 (10 μg/ml) continued to induce LXR and partially SREBP1c, whilst it failed to up-regulate the expression of FAS (Fig. 7D,E, p < 0.05).

### Hepatic expression of LXRα and SREBP1c increases in liver biopsies from HIV patients with NASH

**Figure 8:**
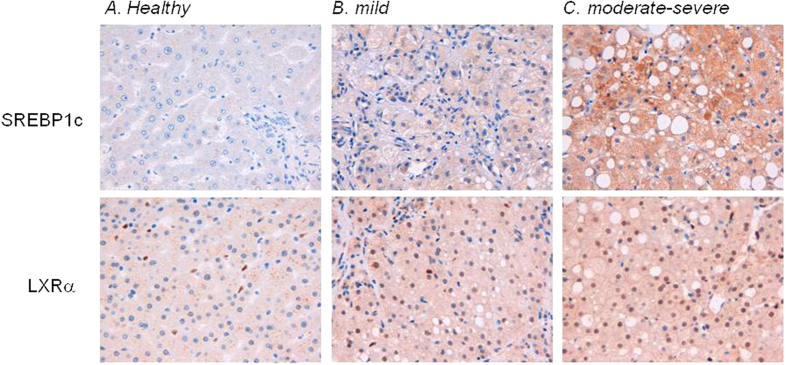
Altered expression of LXR and SREBP1c occurs in hepatic biopsies from HIV infected patients with NASH. Liver biopsies from (**A**) healthy donor, (**B**) HIV infected patient with mild steatosis and (**C**) HIV infected patient with moderate severe hepatic steatosis were serially sectioned and stained with LXRα and SREBP1c antibodies; (Magnification 200x).

Finally, we have investigated the hepatic expression of LXRα and SREBP1c in liver biopsies obtained from HIV monoinfected patients with moderate to severe liver steatosis. As shown in Fig. 8, the immune-histochemical analysis of LXR and SREBP1c reveals that the expression of these proteins, which occurs primarily in hepatocytes, correlated with severity of liver steatosis. Indeed LXR and SREBP1c expression is significantly upregulated in patients with severe steatosis (Fig. 8B,C), thus confirming the *in vitro* observations.

## Discussion

In the present study we have demonstrated that a group of genes encoding for NRs and their coregulators were differentially expressed in HepG2 cells by exposure to p17. Results from microarray analysis demonstrate that nine genes were significantly upregulated (DDX5, NRIP1, ESR1, LXRα, MED1, TR2, TR3, RARα, NCOA1) while six genes were down-regulated (PPARγ, KAT5, BRD8, PSMC5, HDAC7, ARNT) following exposure of HepG2 cells to p17. Noteworthy, among upregulated genes, DDX5 and NRIP1 are multifunctional coactivators of steroid hormone receptors, mainly the Estrogen Receptor (ESR1)^35^,^38^, and are involved in ESR1-mediated adipogenesis^35^. The nuclear receptor LXRα, and its coactivator MED1, have been implicated in central metabolic pathways, including glucose homeostasis, hepatic bile acid metabolism and fatty acid biosynthesis^32^,^39^. Among down-regulated genes, BRD8 and KAT5 are well known PPARγ coactivators and play an essential role in lipid homeostasis, energy metabolism and orchestrate the differentiation of preadipocytes in white, bright or brown adipocytes^33^,^34^,^40^,^41^. Microarray target validation with Real-Time PCR confirmed that the stimulation of HepG2 cells with p17 caused a significant regulation of MED1, LXRα and ESR1 transcripts while failed to regulate the relative mRNA expression of DDX5, BRD8, KAT5 and PPARγ. These results partially provides a molecular explanation to our precedent observations that HIV infection *per se* alters the transcriptome of NRs in monocytes^42^. Consistent with present results, we have previously shown that LXR mRNA levels (but not PPARγ) were significantly induced in monocytes from HIV-infected patients compared to healthy donors, thereby suggesting a direct regulatory role of HIV or HIV proteins in the regulation of this NR^42^.

An important observation we made in this study was that the HIV matrix protein p17 effectively modulated the expression of genes responsive to two nuclear receptors: LXRα and ESR1. Indeed, results from RT-PCR experiments not only confirmed that p17 regulates LXRα target genes such as SREBP1c and CH25H but also the expression of ESR1 responsive genes (i.e. PDK4, PTGDS and PEPCK). Noteworthy, CH25H catalyzes the formation of 25-HC, a LXRα ligand, from cholesterol breackdown^43^,^44^. Analysis of 25-HC and cholesterol content in HepG2 cells reveals that exposure to p17 increases the intracellular content of this lipid mediator without altering total cholesterol content. Taken together, these data illustrate that p17 from one side induces the expression of LXR, and from the other produces a specific ligand for this nuclear receptor, and, ultimately, that these p17-mediated effects translate into a robust lipid accumulation as observed by the Oil red O staining in cells administered p17 for 7 days. Of note, the same regulatory effects exerted by p17 on HepG2 cells, were confirmed in cultures of human and murine primary hepatocytes.

Dissection of intracellular signaling pathway activated by p17 in HepG2 cells demonstrate that the lipogenic effect of p17 is partially due to the activation of the STAT signaling, which in turn enhances the transcriptional activity of LXRα. Consistent with this view we detected a robust activation and recruitment of STAT1 and STAT3 to the LXR promoter after exposure of HepG2 cells to p17. Strengthening this observation, pretreatment of HepG2 cells with STAT1 and STAT3 inhibitors attenuated the regulatory effects of p17 on LXR and LXR-regulated genes.

To provide a clinical readout to our observations we have demonstrated that the serum of an HIV positive patient who underwent a vaccination program with a p17 protein peptide named AT20^14^, effectively neutralizes the lipogenic effects of this viral protein on HepG2 cells. However, in the presence of *gymnestrogenin*, a natural LXR antagonist, the HIV matrix protein p17 continues to partially induce the LXR target gene SREBP1c whilst it failed to induce the FAS, indicating that the LXR/SREBP1c axis is the main, but not the only, mechanism by which p17 exerts its lipogenic effects. These results are consistent with the fact that while the SREBP1c promoter contains responsive elements for LXR and for other NRs whose expression is also modulated by p17 (i.e. PPARs and ER), the FAS promoter contains only responsive motifs for SREBP1c and LXR.

In this work we also evaluated the expression levels of both LXRα and SREBP1c in liver biopsies from HIV infected patients with low to severe steatohepatitis and demonstrated a positive correlation between the levels of immunostaining for LXRα and SREBP1c and the severity of liver steatosis during HIV infection.

In summary, findings presented in this study demonstrated that the HIV-1 matrix protein p17 highjacks STAT pathway causing a derangement of lipid metabolism in hepatocytes. Thus, the presence of circulating p17 particles may contribute to the development of long-term liver dysfunction in HIV infected patients. This study highlights the potential role of anti-p17 based therapies^45^,^46^,^47^ for the treatment of metabolic/liver dysfunction in HIV infection.

## Additional Information

**How to cite this article**: Renga, B. *et al.* The HIV matrix protein p17 induces hepatic lipid accumulation via modulation of nuclear receptor transcriptoma. *Sci. Rep.* **5**, 15403; doi: 10.1038/srep15403 (2015).
